# Some Interesting Cases of Skin and Venereal Disease*Read before Atlanta Society of Medicine, January 4, 1897.

**Published:** 1897-03

**Authors:** Bernard Wolff

**Affiliations:** Atlanta, Ga.


### SOME INTERESTING CASES OF SKIN AND VENEREAL DISEASE.*

BY BERNARD WOLFF, M.D.,

ATLANTA, GA.

CASE 1. Negress; aged 40; café au lait color. General condition poor. History of syphilis several years ago. The eruption for which the patient desired relief on account of the disfigurement consisted of a universal pigmentary syphilide. The duration of the eruption, so far as could be ascertained, was four years, when it appeared shortly after the occurrence of the initial sclerosis. The lesions were maculæ of considerable uniformity of size, being about that of a buckshot; the color was a dingy brown, fairly well defined. There was a slight predilection shown for the classical localities, but the maculæ were numerous upon the face and trunk. There were no subjective symptoms. One-twelfth of a grain of bichloride and five grains of iodide of potash were given three times daily, which showed a favorable influence on the general health, but none upon the lesions themselves. The maculæ have become somewhat less distinct under the persistent use of stimulating ointments.

The pigmentary syphilide must be distinguished from the pigmented relics of ulcerative and degenerative specific lesions, which show a gradual loss of pigment, and ultimately become smooth,

*Read before Atlanta Society of Medicine, January 4, 1897.

slightly depressed, silky, dirty white scars, corresponding to the contour of the original lesion.

The pigmentary syphilide was formerly regarded as rare, but it is now more frequently observed, especially in brunettes and those races subject to pigmentary anomalies, as the Mongolians, Hindus, and Negroes. Pathologically, it is the result of a chronic inflammation of the smaller skin capillaries; the red discs lose their color and finally an obliterating endarteritis imprisons them. This case is presented as interesting on account of the universality of the efflorescence and its unusual persistence.

CASE 2. Morphea; white female, aged 15 years; general health good. Eight months ago the young girl noticed that the skin of the outer side of the dorsum of the left foot was losing its elasticity and pliancy. This was first observed as a patch. The patch gradually elongated, and ascended the limb on the inner side to the groin. Shortly after the skin covering the index-finger became similarly affected and there was a longitudinal upward extension, until the induration faded away gradually on reaching the axilla. The case was referred to me by Dr. H. F. Harris, now of Philadelphia. On examination, I found the affected limbs somewhat smaller than the sound ones. The skin of the outer half of the dorsum of the left foot was tense and glossy, and could not be pinched up. Over the external malleolus there was a deep, punched-out ulcer, surrounded by indurated border. The integument of the anterior tibial region had lost its softness, which had given place to a firm, doughy feeling, but without edema. On the inner side of the knee joint the skin was red and shining, and very inelastic, interfering somewhat with the movement of extension. The skin of the inner side of the thigh, to the groin, was similarly affected. The outer side of the lower extremity, from the lower third of the leg to just above the knee joint was normal. Here there was a strip of hardness two inches across and six long; the color throughout varied from pink to dark brown; here and there, there were small white grouped papules. On the left side of the abdomen, a little below the umbilicus, there were two oval patches of indurated skin as large as the palm of the hand. The color was a faint pink, surrounded by a lilac ring. On the mammary gland below the nipple, there was an elongated patch, firm to the touch, smooth and polished on the surface. The left cheek showed a smooth white patch, with a lilac ring of dilated capillaries surrounding it.

The index-finger of the left hand was semi-flexed, held in this position by the rigidity of the skin. The skin of the inner side of the forearm and the arm showed the same changes as noted in the lower extremity. The pigmentation was the same.

A section of skin was removed from the forearm for the purpose of examination pathologically, and the wound healed by granulation in a few weeks.

There was complete anidrosis of the affected areas. There were no subjective symptoms except those incident upon the curtailment of movement by the rigidity of the skin. The disease has become steadily and progressively worse. The bands of infiltration have become harder, more rigid, and the limbs from disease have become atrophied to a startling extent when compared with those of the sound side. The left lower extremity is flexed at the knee joint, at an angle of 45 degrees, rendering locomotion very difficult without the aid of a crutch. A recent attack of la grippe has greatly aggravated the symptoms.

Treatment has so far been of no avail. Thyroid extract (Park, Davis & Co.), together with ten grains of potassium iodide, was given for two months in doses of 25 grains per diem. It was finally withdrawn on account of producing distressing palpitation of the heart and insomnia.

Morrow has recently reported a case, which, at the time of writing, was rapidly improving under similar treatment. Perhaps the gods were kinder to him than to me. Local treatment consisted first in stimulating inunction with salves containing 10-20 per cent. salicylic acid and resorcin. The skin becoming swollen and excoriated and no benefit being apparent, this form of treatment was abandoned for bland ointment of ichthyol and adeps lauae, accompanied with massage. Iodide of potash was administered by cataphoresis without improvement. All efforts at specific treatment have now been discontinued, and tonic and supportive measures adopted, as massage, salt baths, with elixir of strychnine, iron and quinine.

Morphea or Addison's keloid is an extremely rare affection, as is proved by the circumstance that the statistics of the American Dermatological Association record but two instances among 16,863 cases of skin disease. McCall Anderson found two cases of morphea among ten of scleroderma in 40,000 cases of skin disease. It is generally unilateral, prone to occur in elongated patches, which follow the course of superficial nerves. The patches are not elevated. When the patch is very white, it is called macula alba; when much pigmented, macula nigra; when dry and parchment-like, macula atrophica. The disease is regarded as a thickening and stasis of lymph in the cutis, due not to local changes, but alterations in the general nutritive processes. When the lymph channels become free the infiltration disappears. If the stagnation be of long duration, out of the superfluity of nutrient material, connective tissue is formed in excess and becomes denser and increased in quantity, and contract. The prognosis is generally favorable. The rule is, disappearance of all infiltration and return of the skin to the normal, leaving no trace of the disease after a varying lapse of time.

Case 3. Negro; 30 years of age; specific history. On the outer side of the dorsum of both feet, there was a placque the size of a dollar piece. Around each there were several flattened papules; the placques were elevated, sodden, dirty white, resembling a condyloma lata, but lacking its moisture. The sodden epidermis, when removed, disclosed a faintly reddened surface. This variety of papular syphilide is termed corymbiform from its resemblance to a flower cluster, in which the axis is somewhat shortened, and the pedicles of the lower flowers are so lengthened as to form a flat-topped cluster.

Case 4. Ainhum. Colored woman; 40 years old; born in this State and has lived here all her life; family history negative. About twenty years ago a constriction formed at the digito-plantar fold of the right little toe. The constricting band slowly narrowed until the toe was almost severed from the foot. It was then removed by a surgeon. About ten years ago, a similar constricting band appeared at the same place on the left side. The narrowing of the ring progressed slowly but surely. She applied for aid on account of the pain from a small ulcer between the little and next toes, and from the inconvenience caused by it in walking. I found the toe connected with the foot by a narrow ligament, the toe itself transformed into a globular fatty mass, with a thin strip of curled up nail in the usual position. It was freely movable in all directions. I removed the toe with scissors. Microscopically, it resembled a polyp, round, smooth, with a pedicle which represented the phalanx. There was scarcely a trace of osseous tissue remaining. The bone has been transformed into fibrous tissue, in which fat was freely mingled. I sent the toe to Dr. T. C. Gilchrist of Johns Hopkins Hospital, Baltimore, and expect with him to make an extended report of the case.

Ainhum was first described by Da Silva Lima, of Bahia, Brazil, in 1867. The name is derived from a native word meaning "to saw." The geographical distribution is wide, but it has never been observed in a white person. A large number of cases have been recorded in India, Ceylon and China; the Polynesian islands have produced instances of the disease. It is well known in Africa, especially Egypt, the west coast, the South African republic and the interior. In fact, the disease was first known, before described by Lima, as dry gangrene of the gold coast.

The disease is found to occur in the West Indies, South America and in North America; cases have been observed in Louisiana, North Carolina, Pennsylvania, South Carolina and Canada. So far as I know, this is the first case reported in this State, though doubtless other cases have been seen.

The disease having a racial restriction would seem to be due to some peculiar predisposition on the part of negroes and negroid peoples to it. The habit of going barefooted, of wearing toe rings and of foreign bodies between the toes, have been suggested as causes of the trouble, but all fall far short of offering even a plausible explanation. To me the possibilities of flat foot, as a causative factor, have been overlooked. This condition is extremely frequent among those subject to ainhum, and not much consideration of the mechanical effects of the deformity, with which I shall not occupy your time, will lead one to the belief that this theory offers at least a more reasonable explanation of this very curious phenomenon than any hitherto advanced.

